# Acetylcholine receptor antibody-positive myasthenia gravis and Kennedy’s disease overlap syndrome: a case report and literature review

**DOI:** 10.3389/fimmu.2026.1872718

**Published:** 2026-06-11

**Authors:** Guo-hui Gao, Yu-qing Xu, Yu-dong Liu, Ying Liu, Jing Yuan

**Affiliations:** 1 Department of Neurology, The First Affiliated Hospital of Shandong First Medical University &Shandong Provincial Qianfoshan Hospital, Jinan, China; 2 Shandong Institute of Neuroimmunology, Jinan, China; 3 Shandong Provincial Medicine and Health Key Laboratory of Neuroimmunology, Jinan, China

**Keywords:** androgen receptor gene, Kennedy’s disease, myasthenia gravis, overlap syndrome, spinal and bulbar muscular atrophy

## Abstract

**Objective:**

To investigate the clinical characteristics and electromyographic findings of a patient with acetylcholine receptor (AChR) antibody-positive myasthenia gravis (MG) overlapping with Kennedy’s disease (KD), aiming to improve clinicians’ recognition of this rare overlap syndrome and explore potential comorbid mechanisms.

**Methods:**

The clinical presentation, laboratory results, and neurophysiological features of one case with AChR antibody-positive MG and KD were analyzed. A literature review of MG overlapping with KD was performed.

**Conclusion:**

Neurophysiological findings and androgen receptor gene testing results were consistent with a diagnosis of KD. Therefore, the patient was definitively diagnosed with overlap syndrome of AChR antibody-positive MG and KD. A literature review identified only four previously reported cases of KD patients exhibiting myasthenic features; however, all were AChR antibody-negative.

## 1 Introduction

Myasthenia gravis (MG) is an autoimmune disease mediated by autoantibodies targeting components of the postsynaptic membrane at the neuromuscular junction (NMJ). Its primary clinical feature is fluctuating weakness of skeletal muscles.

Among these, anti-acetylcholine receptor (AChR) antibodies are the most common pathogenic antibodies. Diagnosis relies on typical clinical manifestations, serum antibody detection, a decrement response on low-frequency repetitive nerve stimulation (RNS), and a positive therapeutic response to cholinesterase inhibitors, with relevant criteria clearly defined by international consensus guidelines (1).

Kennedy’s disease (KD), also known as spinal and bulbar muscular atrophy, is a rare X-linked recessive motor neuron disorder (2).The disease is caused by an abnormal expansion of CAG trinucleotide repeats, which encode polyglutamine, in the androgen receptor (AR) gene; the repeat length is inversely correlated with the age of onset (3). Its clinical features include progressive bulbar and limb muscle weakness and atrophy, gynecomastia, and sensory neuropathy. Neurophysiologically, it is characterized by widespread neurogenic changes.

The coexistence of MG and KD in the same patient is exceptionally rare. Here, we present the first case of genetically confirmed KD with concomitant AChR antibody−positive MG. Through detailed case description and literature review, we aim to enhance recognition of this overlap syndrome and discuss possible comorbid mechanisms.

## 2 Methods

### 2.1 Clinical data

Detailed clinical assessments, neurophysiological evaluations, and genetic analyses were performed on the patient. The study protocol received ethical clearance from the Ethics Committee of the First Affiliated Hospital of Shandong First Medical University. Prior to enrollment, the patient gave written informed consent for participation.

### 2.2 Assessment of nerve and NMJ disorders by electromyography

Neuroelectrophysiological assessments primarily included the use of a Nihon Kohden MEB-9400 EMG machine (Japan) to evaluate NMJ function and the extent and nature of nerve damage. Surface electrodes were used for nerve conduction studies and repetitive nerve stimulation (RNS). A concentric needle was used to perform needle electromyography (EMG) to observe abnormal spontaneous potentials (positive sharp waves and fibrillation potentials) and the discharge patterns (duration, activation, recruitment, and interference patterns) of motor unit action potentials (MUAPs).

### 2.3 Genetic testing

The number of CAG repeats in exon 1 of the AR gene was detected by polymerase chain reaction (PCR) and capillary electrophoresis (Simcere Dx Co., Ltd.). The normal reference range was ≤34.

### 2.4 Literature review

The PubMed database was searched using the terms “Kennedy’s disease”, “spinal and bulbar muscular atrophy”, “myasthenia gravis”, and “myasthenic weakness”, without language or publication date restrictions. Four case reports of KD patients presenting with myasthenic symptoms that met the clinical and electrophysiological diagnostic criteria for MG were identified (**Table 1**) (4–7). The clinical features, EMG findings, and laboratory results of the present case were summarized.

**Table 1:** Summary of reported cases with features of both myasthenia gravis and Kennedy’s disease.

Case (Reference)	Sex/Age	Disease duration	Clinical presentation									
			Bulbar symptoms	Motor symptoms	Fluctuation	Fasciculations	Sensory symptoms	Endocrine features	Muscle atrophy	Muscle strength	Tendon reflexes	Pathologic reflexes
P1 (Yamada et al., 1997)	M/60	13 y	(-)	Ptosis, Proximal weakness	Yes (Fatigue)	Tongue	(-)	Testicular atrophy	Tongue, Face, Proximal	NA	NA	NA
P2 (Boz et al., 2004)	M/34	6 mo	Dysphagia	Diplopia, Ptosis	Yes (Diurnal)	Tongue	(-)	Gynecomastia, Testicular atrophy	Testes	Mild facial/neck weakness	Reduced (Absent ankle)	(-)
P3 (Stević et al., 2014)	M/49	5 y	Dysphagia	Diplopia, Ptosis, Fatigue	Yes (Afternoon)	Face, Tongue, Limbs	NA	Gynecomastia	Tongue, Trunk, Limbs	NA	Absent	NA
P4 (Jamora et al., 2021)	M/51	30 y	Dysphagia, Dysarthria	Ptosis, Proximal weakness	Yes (Evening/Post-exertion)	Bulbar, Cervical, Lumbosacral	(-)	Gynecomastia, Testicular atrophy	Tongue, Testes	Proximal 4/5, Distal 5/5	Reduced	NA
P5 (Our Case, 2025)	M/60	4 y	Dysarthria, Choking on liquids.	Ptosis (Fatigable), Proximal upper limb weakness	Yes	Tongue	(-)	Gynecomastia	Tongue	Proximal UE 3/5, Distal/LE 5/5	Reduced	(-)
Case (Reference)	Neurological examination investigations									Treatment & genetics		
	NCS		Needle EMG		NMJ Studies			Serum CK	MG-related antibodies	Effective therapy	AR Gene CAG repeats	
	Motor	Sensory	Spontaneous activity	MUP / recruitment	RNS (decrement)	SFEMG	Muscle biopsy					
P1 (Yamada et al., 1997)	Normal	Normal	NA	Large, polyphasic MUAPs; Reduced recruitment	Positive (5 Hz)	NA	NA	2014 U/L	AChR (-)	Pyridostigmine	45	
P2 (Boz et al., 2004)	Normal	Reduced SNAP amplitude	Present (Tongue, SCM)	Large, long-duration, polyphasic MUAPs	Positive (3 Hz)	Increased jitter, Blocking	NA	322 U/L	AChR (-)	Pyridostigmine	49	
P3 (Stević et al., 2014)	Normal	Normal	NA	Large, polyphasic MUAPs	Positive (post-fatigue)	Increased jitter/blocking (10 Hz)	NA	Normal	AChR (-), MuSK (-)	Pyridostigmine, Steroids	48	
P4 (Jamora et al., 2021)	Normal	Normal	NA	Large, polyphasic, unstable MUAPs; Reduced recruitment	Positive (4 Hz)	Increased jitter, Blocking	Large group atrophy, No inflammation	479 U/L	Negative	Pyridostigmine, Steroids	47	
P5 (Our Case, 2025)	Normal	Reduced SNAP amplitude	Present (Rectus abdominis, Deltoid, TA)	Large, polyphasic MUAPs; Reduced recruitment	Positive(3Hz)	Increased jitter, Blocking	NO	605 U/L	AChR (+)	Pyridostigmine, Steroids	39	

M, Male; y, years; mo, months; NA, Not available; CK, Creatine Kinase; AChR, Acetylcholine Receptor; NCS, Nerve Conduction Studies; SNAP, Sensory Nerve Action Potential; EMG, Electromyography; SFEMG, Single-Fiber Electromyography; UE, Upper Extremity; LE, Lower Extremity; TA, Tibialis Anterior; SCM, Sternocleidomastoid. MUAPs, Motor Unit Potential; RNS, Repetitive Nerve Stimulation.

## 3 Results

### 3.1 Case report

A 60-year-old man developed diplopia and bilateral ptosis approximately 10 days after an episode of frequent diarrhea in 2021. The symptoms exhibited diurnal fluctuation, worsening with fatigue and improving with rest. Serological testing at an external hospital revealed strongly positive AChR antibodies (>20 nmol/L), and a neostigmine test was positive, meeting the diagnostic criteria for MG. Since disease onset, his condition has fluctuated and worsened repeatedly due to triggers such as “staying up late, alcohol consumption, emotional excitement, fatigue, and infections,” leading to multiple hospital visits and irregular treatments. Initial treatment consisted of pyridostigmine 60 mg three times daily combined with prednisone 40 mg once daily. After 1–2 weeks of treatment, ocular symptoms markedly improved. The patient was discharged after stabilization, after which prednisone was tapered by 5 mg every 2 weeks to a maintenance dose of 10 mg once daily. In December 2021, the patient experienced disease progression with new-onset dysphagia and choking on water. Intravenous methylprednisolone pulse therapy (500 mg once daily for 3 days) was administered, followed by oral prednisone 30 mg once daily with gradual tapering according to clinical response. Swallowing difficulty showed short-term improvement. In July 2022, one year after disease onset, the patient developed weakness in all four limbs accompanied by fasciculations in the lower limbs. He received intravenous immunoglobulin (IVIG) 0.4 g/kg once daily for 5 days, oral prednisone 20 mg once daily, and pyridostigmine was adjusted to 60 mg every 6 hours. Limb weakness partially improved, but fasciculations persisted. Subsequently, the patient experienced another exacerbation. In May 2023, plasma exchange was administered every other day for a total of 5 sessions, resulting in short-term improvement of limb weakness and dysphagia lasting approximately one month.

**Figure 1:**
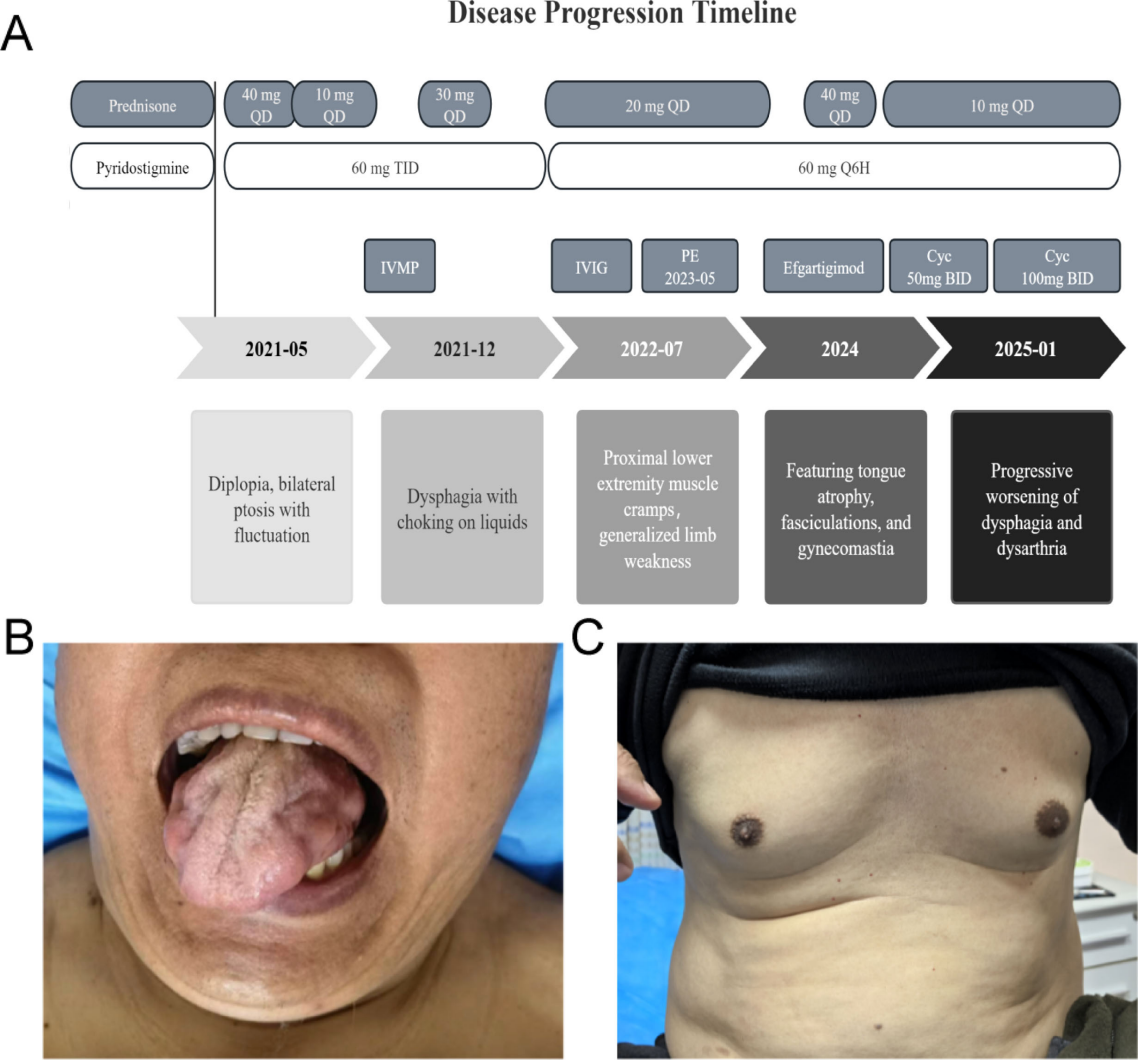
**(A)** Disease progression timeline. QD, once daily; BID, twice daily; TID, three times daily; Q6H, every 6 hours; IVMP, intravenous methylprednisolone pulse therapy; IVIG, intravenous immunoglobulin; PE, plasma exchange; Cyc, cyclophosphamide. **(B)** Tongue atrophy. **(C)** Gynecomastia.

In 2024, the patient’s condition further deteriorated. Physical examination revealed tongue atrophy with fasciculations and gynecomastia, along with progressive limb weakness. Efgartigimod alfa was administered for a total of 6 cycles, during which the patient’s condition stabilized, and prednisone was adjusted to 40 mg once daily. Prednisone was then tapered by 5 mg every 2 weeks. In September 2024, the patient experienced another symptomatic exacerbation. Oral cyclophosphamide was added at an initial dose of 50 mg twice daily, which was gradually increased to 100 mg twice daily based on tolerance, resulting in clinical improvement. During the last four months before admission (January 2025 to admission), dysphagia and dysarthria progressively worsened. The patient regularly took pyridostigmine 60 mg every 6 hours, cyclophosphamide 100 mg twice daily, and prednisone 10 mg once daily, without significant relief. For further systematic evaluation and management, the patient was admitted on April 1, 2025 (**Figure 1**). His past medical history included hypertension for 3 years and a history of lacunar infarctions for many years without sequelae. He has one son and one daughter; family history was unremarkable.

Physical examination revealed gynecomastia, weak cheek puffing, dysarthria, dysphagia, choking on water, tongue fasciculations and atrophy. Neck flexion strength was Medical Research Council grade 3, bilateral proximal upper limbs grade 4, bilateral distal upper limbs and bilateral lower limbs grade 5. Sensory examination was normal. Deep tendon reflexes were absent. Palmomental reflex and pathologic reflexes were negative. Finger-to-nose and heel-to-shin tests were negative, as were meningeal irritation signs.

Initial routine blood tests revealed mildly decreased leukocytes, neutrophils, erythrocytes, and hemoglobin. Glycated hemoglobin was 6.2%, and postprandial blood glucose levels were elevated. Liver function tests revealed mild abnormalities (ALT 87.6 U/L, AST 63.9 U/L). Serum creatine kinase was elevated to 605 U/L. All other routine and immunological parameters were within normal limits. Repeat testing for myasthenia gravis-associated antibodies showed anti−AChR IgG > 20 nmol/L, while antibodies against muscle−specific kinase (MuSK), low−density lipoprotein receptor−related protein 4 (LRP4), ryanodine receptor (RyR), and titin were all negative. Neuroimaging was performed to evaluate for intracranial lesions and thymic abnormalities. Brain CT showed minor ischemic foci and mild craniocervical atherosclerosis. Chest CT identified a right pulmonary nodule, with no evidence of thymoma or thymic hyperplasia.

### 3.2 Electromyography

On April 2, 2025, electrophysiological studies were performed. Low-frequency RNS tests at 3 Hz on the right accessory, right facial, and left median nerves showed a decremental response in compound muscle action potential amplitude exceeding 10%. Specifically, the decrement was 13.7% on the right accessory nerve, 11% on the right facial nerve, and 12.8% on the left median nerve. Needle electromyography was conducted in multiple muscles, including the left extensor digitorum communis, left first dorsal interosseous, left deltoid, left trapezius, left rectus abdominis, tongue, right extensor digitorum communis, right extensor pollicis brevis, right tibialis anterior, and right quadriceps femoris, et al. At rest, fibrillation potentials and positive sharp waves were seen in the left deltoid, left rectus abdominis, and right tibialis anterior, indicating active denervation. During low−force contraction, MUAPs in all examined muscles showed increased duration, increased amplitude, and polyphasic morphology. Quantitation analysis of the right quadriceps femoris based on motor unit action potentials revealed a prolonged mean duration of 17.9 milliseconds, and an increased mean amplitude of 3824 microvolts. During high−force contraction, all examined muscles displayed reduced recruitment. F−wave studies showed markedly reduced persistence of the median nerves, with 30% on the right and 50% on the left. The right ulnar nerve exhibited giant F−waves characterized by abnormally increased amplitude. Sensory nerve conduction studies revealed absent sensory nerve action potentials in the median and ulnar nerves bilaterally, as well as reduced conduction velocities in the superficial peroneal and sural nerves.

### 3.3 Genetic testing

Peripheral venous blood was collected for AR gene fragment analysis. The results revealed abnormal expansion of CAG trinucleotide repeats in exon 1 of the AR gene, with a repeat length of approximately 39 (abnormal >34).

### 3.4 Diagnosis

Based on clinical presentation and ancillary examinations, the patient was diagnosed with overlap syndrome of AChR antibody-positive MG and KD, accompanied by elevated transaminases.

## 4 Discussion

**Figure 2:**
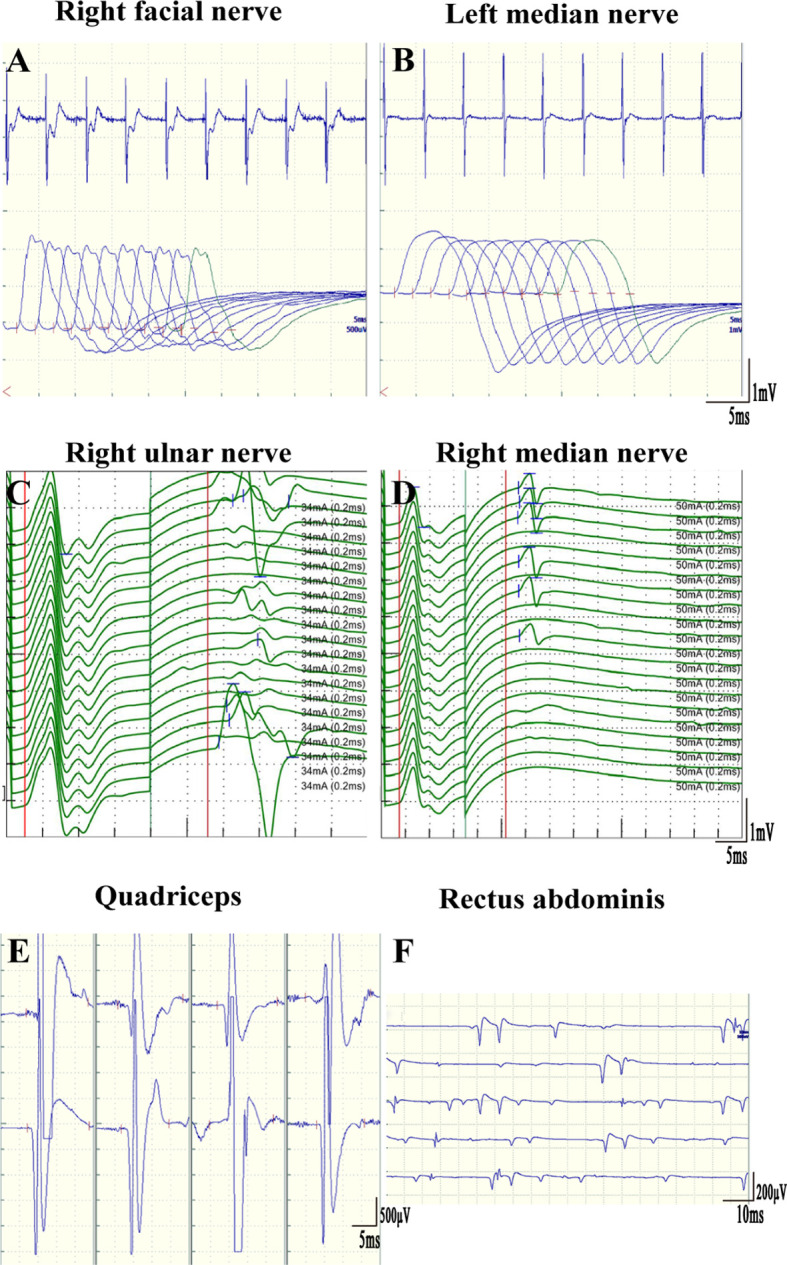
Neurophysiological investigations. **(A, B)** Low-frequency (3 Hz) repetitive nerve stimulation showing decremental responses in the right facial and left median nerves. **(C, D)** F-wave studies of the right median and ulnar nerves. The right median nerve shows severely reduced persistence, while the right ulnar nerve demonstrates giant F-waves. **(E)** Needle EMG of the right quadriceps demonstrating motor unit potentials with prolonged duration and increased amplitude. **(F)** Spontaneous activity in the left rectus abdominis muscle.

The patient’s initial presentation with typical fluctuating weakness, strongly positive AChR antibodies, a decrement on low-frequency RNS, and response to cholinesterase inhibitors fulfilled the diagnostic criteria for MG. However, as the disease progressed, the development of progressive tongue fasciculations and atrophy(**Figure 1B**), along with gynecomastia(**Figure 1C**), represented features not explained by typical MG. Furthermore, the poor response to intensified immunotherapy suggested the possibility of a coexisting condition. EMG confirmed widespread neurogenic changes, predominantly chronic reinnervation: giant F-waves(**Figures 2C, D**), large polyphasic motor unit action potentials (MUAPs) (**Figures 2E, F**), along with spontaneous activity (fibrillation potentials), pointing to an anterior horn cell disorder, ultimately confirmed as KD by genetic testing.

Ptosis and diplopia are the most common initial symptoms of MG, followed by involvement of facial, bulbar, limb, and respiratory muscles. In this patient, the involvement of specific muscle groups, marked symptom fluctuation, strongly positive serum AChR antibodies (>20 nmol/L), a significant decrement on low-frequency RNS (>10%)(**Figures 2A, B**), a positive neostigmine test, and sensitivity to cholinesterase inhibitors all aligned with the diagnostic criteria for MG.

The core molecular mechanism of KD involves the formation of toxic nuclear inclusions from mutant androgen receptor protein aggregates in motor neurons, leading to transcriptional dysregulation and progressive degeneration of lower motor neurons (8). Inclusions are found not only in spinal and bulbar motor neurons, causing weakness, atrophy, and bulbar symptoms (dysarthria, dysphagia, choking), but also in non-motor neurons, contributing to non-motor manifestations such as sensory neuropathy and endocrine abnormalities (9). Additionally, decreased AR function results in partial androgen insensitivity syndrome (P-AIS), characterized by androgen insufficiency symptoms like gynecomastia and sexual dysfunction (10). This patient presented with adult-onset, slowly progressive bulbar and proximal limb weakness, accompanied by tongue atrophy, fasciculations, and gynecomastia; elevated serum CK; EMG findings of widespread neurogenic damage; and genetic confirmation of KD.

The patient exhibited marked tongue atrophy and fasciculations. Tongue atrophy is rare in MG and typically does not occur with fasciculations. In MuSK-MG, tongue atrophy is a distinct feature, often accompanied by involvement of other bulbar muscles, including the masseter and facial muscles (11). Although tongue atrophy is more common in anti-MuSK MG than in AChR-MG, the patient tested negative for anti-MuSK antibodies, suggesting that a purely myasthenic cause is less likely. The underlying mechanism involves IgG4 autoantibodies that block MuSK-LRP4 interaction. This impairs agrin-induced acetylcholine receptor clustering and causes postsynaptic instability; chronic NMJ disorganization may eventually lead to muscle fiber atrophy (12). Zhao et al. (2024) reported six MG cases with tongue atrophy, including anti−AChR MG, anti−MuSK MG, and seronegative MG. Tongue EMG in those cases showed myopathic changes, and the atrophy was usually reversible with effective immunotherapy (13), which differentiates it from the neurogenic tongue atrophy observed in KD. In this patient, tongue atrophy progressed despite continuous immunosuppressive therapy, indicating an additional neurological disorder beyond MG.

Further neurophysiological examination provided crucial diagnostic information. Previous research indicates that the decrement pattern in MG on RNS is typically “U-shaped”, where the amplitude reaches its nadir at the 4th or 5th response and then shows significant recovery from the 6th-7th response onwards due to replenishment of the readily releasable pool of synaptic vesicles. In this patient, 3-Hz RNS of the right facial and ulnar nerves showed a significant decrement (>10%), indicative of NMJ transmission defect. However, the waveform morphology and subsequent recovery pattern appeared “L-shaped”, differing from the classic “U-shaped” pattern of MG. This L-shaped decrement pattern can also be observed in other anterior horn cell disorders, such as amyotrophic lateral sclerosis (ALS). ALS, characterized by progressive degeneration and loss of motor neurons in the spinal cord and brainstem, is a prototypical anterior horn cell disease. Fu et al. (2019) analyzed RNS waveform trends in ALS and supported that different etiologies lead to distinct transmission defect patterns, showing that the low-frequency RNS decrement curve in ALS patients is often “L-shaped”, with little to no recovery after the nadir (14). Iwanami et al. (2011) suggested that decrements in motor neuron diseases might arise from different physiological bases: following NMJ structural damage, surviving lower motor neurons around degenerating neurons may reinnervate denervated muscle fibers through collateral sprouting, forming new, immature NMJs. This could lead to reduced ACh release and storage, lowering the safety factor of neuromuscular transmission (15). We hypothesize that in this patient, chronic, long-term loss of anterior horn cells due to the genetic defect resulted in an RNS decrement pattern similar to that seen in ALS patients, fundamentally different from the mechanism in typical MG where autoantibodies attack the postsynaptic membrane. Nerve conduction studies revealed sensory fiber involvement. Although the patient had abnormal glucose metabolism, the EMG findings of sensory fiber involvement were non-length dependent and widespread, not fitting the pattern of diabetic peripheral neuropathy, thus pointing to other causes. Sensory fiber involvement in KD primarily results from pathology in sensory neurons. More diagnostically valuable were the F-wave findings. Giant F-waves are considered a hallmark of chronic anterior horn cell loss, reflecting compensatory hyperexcitability of surviving motor neurons and axonal collateral sprouting, leading to enlarged motor units (16). In this patient, EMG recorded F-waves with reduced persistence, abnormally increased amplitude, and increased duration (giant F-waves) in multiple motor nerves, a characteristic electrophysiological marker of KD. Furthermore, needle EMG revealed widespread spontaneous activity (fibrillation potentials, positive sharp waves) at rest. When muscle fibers lose their nerve supply, their membrane electrophysiological properties change, becoming unstable and prone to spontaneous depolarization. Spontaneous activity is rare in pure NMJ disorders, except in conditions like botulism. Large, long-duration, polyphasic MUAPs arise from compensatory collateral sprouting of surviving neurons following motor neuron loss, forming enlarged motor units – a classic chronic neurogenic change in KD. In this patient, muscles of the limbs and trunk showed MUAPs with significantly increased duration and amplitude, and reduced recruitment with a simple pattern during maximal contraction. This pattern indicates extensive chronic neurogenic damage, with co-existing acute denervation potentials (fibrillations) and chronic reinnervation signs (large MUAPs), consistent with the long-term, slowly progressive pathophysiology of KD. This contrasts sharply with the small, short-duration, polyphasic MUAPs often seen in MG due to loss of individual muscle fiber action potentials.

The clinical and laboratory evidence above strongly suggests that this patient has both MG and KD. We summarized the clinical features of this case alongside previously reported cases (**Table 1**), These cases all exhibited features like fluctuating weakness, decrement on low-frequency RNS, and response to cholinesterase inhibitors. However, serum AChR and muscle-specific tyrosine kinase (MuSK) antibodies were negative in all four previously reported patients. This led to differing interpretations of the myasthenic weakness: Yamada suggested it might be a “myasthenic syndrome” resulting from chronic denervation and aberrant reinnervation inherent to KD, rather than an independent autoimmune disorder. Boz, however, proposed coexistence of KD and seronegative MG as two distinct diseases. In stark contrast to all prior reports, the present case is the first genetically confirmed KD patient with concomitant AChR antibody-positive MG. The strongly positive serum AChR antibody (>20 nmol/L) provides definitive serological evidence supporting the rare coexistence of two independent diseases.

We next compared our findings with previously reported overlaps between MG and muscular dystrophy (MD). Although the coexistence of MG and MD is rare, it has been systematically characterized. Avallone et al. (17) identified 20 cases of anti-AchR antibody-positive MG overlapping with various MDs, with facioscapulohumeral muscular dystrophy being the most common subtype (35% of cases). In their cohort, the mean AChR antibody titer was 22.3 nmol/L, and 80% of patients presented with generalized MG. Repetitive nerve stimulation yielded positive results in 90% of tested cases, highlighting its diagnostic value. In contrast, the overlap between MG and KD is far rarer, with only four cases reported to date. While the high AChR antibody titer and generalized MG phenotype in our patient resemble those seen in MG–facioscapulohumeral muscular dystrophy overlap, the presence of endocrine manifestations, neurogenic electromyographic changes, and X-linked inheritance clearly distinguishes MG–KD overlap from other MG–MD associations.

KD may disrupt NMJ stability and affect the entire motor unit from the motor neuron to the muscle membrane through multiple mechanisms, potentially providing a pathological basis for MG development or exacerbating immune responses. Proposed mechanisms include: 1. Direct synaptic toxicity: Mutant AR in KD may directly impair NMJ function. Studies show that AR is normally enriched in myonuclei at the NMJ, and its expression correlates with muscle androgen sensitivity. AR may act as a transcription factor regulating synaptic-specific genes in motor neurons. Accumulation of mutant AR at the NMJ could exert direct toxic effects, impairing postsynaptic membrane stability and neuromuscular transmission efficiency (18). 2. Muscle involvement: Most KD patients have elevated CK levels (19), and muscle biopsies often show myopathic changes (20), indicating pathology is not confined to motor neurons but also directly affects muscle fibers. These chronic, ongoing processes of neuronal death and NMJ damage release cellular debris and autoantigens, potentially altering the local microenvironment and breaking immune tolerance, thereby providing a substrate for secondary autoimmune responses that could trigger or exacerbate autoimmunity against synaptic components like AChR (21).

Similarly, in ALS, another anterior horn cell disease characterized by chronic, progressive motor neuron loss, disruption of the normal immune environment may potentially facilitate the superimposition of MG. Tai et al. (2017) reviewed evidence of widespread immune activation and dysregulation in ALS patients, including regulatory T-cell dysfunction and elevated pro-inflammatory cytokines (22). The continuous release of neuronal and NMJ components during neurodegeneration could initiate specific autoimmune responses against NMJ structural proteins (e.g., AChR, LRP4, MuSK). Studies by Ashizawa (23) and Okuyama et al. (24) showed that about 5% of ALS patients have detectable low-titer AChR antibodies without clinical MG, with titers sometimes fluctuating with disease activity, suggesting antibody production correlates with the intensity of motor unit damage or NMJ stress. In a subset of patients, this secondary antibody response might reach a threshold sufficient to directly attack and impair NMJ function, resulting in superimposed clinical MG. However, why only a very small fraction of patients with anterior horn cell diseases develop overt MG, and the key genetic or environmental factors driving this process, require further investigation.

## 5 Conclusion

Although MG and KD belong to different disease categories, they can co-occur in the same patient. Clinicians should maintain a high index of suspicion for KD in MG patients presenting with tongue atrophy. Systematic neurophysiological evaluation, detailed physical examination, and genetic testing are crucial for identifying such overlap syndromes and avoiding misdiagnosis.

## Data Availability

The original contributions presented in the study are included in the article/supplementary material. Further inquiries can be directed to the corresponding authors.
